# MRI as a Novel In Vivo Approach for Assessing Structural Changes of *Chlamydia* Pathology in a Mouse Model

**DOI:** 10.1371/journal.pone.0160055

**Published:** 2016-07-28

**Authors:** Catherine D. G. Hines, Shubing Wang, Xiangjun Meng, Julie M. Skinner, Jon H. Heinrichs, Jeffrey G. Smith, Melissa A. Boddicker

**Affiliations:** 1 Department of Translational Imaging Biomarkers, MRL (West Point, PA), Merck & Co., Inc., Kenilworth, New Jersey, United States of America; 2 Department of Biometrics Research, MRL (Rahway, NJ), Merck & Co., Inc., Kenilworth, New Jersey, United States of America; 3 Department of Vaccines Early Discovery, MRL (West Point, PA), Merck & Co., Inc., Kenilworth, New Jersey, United States of America; 4 Global Project Leadership, Sanofi Pasteur, Swiftwater, Pennsylvania, United States of America; 5 Pharmaceutical Sciences Analytical Research & Development, Pfizer, Andover, Massachusetts, United States of America; Univ. of Texas Health Science Center at San Antonio, UNITED STATES

## Abstract

*Chlamydia trachomatis* is among the most prevalent of sexually transmitted diseases. While *Chlamydia* infection is a reportable event and screening has increased over time, enhanced surveillance has not resulted in a reduction in the rate of infections, and *Chlamydia* infections frequently recur. The development of a preventative vaccine for *Chlamydia* may be the only effective approach for reducing infection and the frequency of pathological outcomes. Current vaccine research efforts involve time consuming and/or invasive approaches for assessment of disease state, and MRI presents a clinically translatable method for assessing infection and related pathology both quickly and non-invasively. Longitudinal T2-weighted MRI was performed over 63 days on both control or *Chlamydia muridarum* challenged mice, either with or without elementary body (EB) immunization, and gross necropsy was performed on day 65. A scoring system was developed to assess the number of regions affected by *Chlamydia* pathology and was used to document pathology over time and at necropsy. The scoring system documented increasing incidence of pathology in the unimmunized and challenged mice (significantly greater compared to the control and EB immunized-challenged groups) by 21 days post-challenge. No differences between the unchallenged and EB immunized-challenged mice were observed. MRI scores at Day 63 were consistently higher than gross necropsy scores at Day 65, although two of the three groups of mice showed no significant differences between the two techniques. In this work we describe the application of MRI in mice for the potential evaluation of disease pathology and sequelae caused by *C*. *muridarum* infection and this technique’s potential for evaluation of vaccines for *Chlamydia*.

## Introduction

*Chlamydia trachomatis* is the most commonly reported disease in the United States and among the most prevalent of sexually transmitted diseases. Approximately 1.4 million cases were reported to the Centers for Disease Control and Prevention in 2012, and the incidence rate was 457 out of 100,000 individuals [1]. From 1999–2008, the prevalence of *Chlamydia* infection in sexually active females from 14–19 years of age was 6.8% [2]. While *Chlamydia* infection is a reportable event and screening has increased over time, enhanced surveillance has not resulted in a reduction in the rate of infections [3].

*Chlamydia* infection causes intense inflammation at the mucosal site of infection and can result in the clinical conditions of urethritis or mucopurulent cervicitis [4]. Many urogenital infections are asymptomatic, and as a result, many infections remain undiagnosed and therefore go untreated until complications develop. Ascending *Chlamydia* infection can lead to additional chronic disease sequelae that may include pelvic inflammatory disease (PID), ectopic pregnancy, or even tubal factor infertility (TFI) [5, 6]. In addition, recurrent infections occur in up to 30% of women and may increase the risk of complications, and antibiotic treatment may enhance the recurrence of infection by limiting development of protective immune responses [7].

Early time points following genital tract infection with *Chlamydia* in animal models and humans are marked by an inflammatory response and cytokine release including TNFα, IL-8, IL-1α, and GM-CSF [4, 8]. Mice infected with *Chlamydia muridarum* exhibit an influx of neutrophils, dendritic cells, T- and B-cells and macrophages in the genital tract tissue [9]. Inflammation is a part of the innate arm of the immune system and is an initial response to pathogen infection. Inflammation is characterized by heat, pain, redness, swelling, and loss of tissue function. It is the early inflammatory response elicited by *Chlamydia* infection that can result in tissue destruction, fibrosis and scarring. As observed by de La Maza, oviducts showed marked dilation and atrophy of the mucosal layer by day 37 and 49 upon histological examination [10].

The development of a preventative vaccine for *Chlamydia* may be the only effective approach to reduce infection and the frequency of pathological outcomes. Current research efforts have focused on screening antigens and evaluating experimental vaccines. Infection with *C*. *muridarum* in mice results in a virulent infection that ascends to the upper genital tract and results in tubal scarring and infertility [5]. The mouse models of *C*. *muridarum* urogenital infection have proven useful for vaccine evaluation [11–15]. In these models, both the level of vaginal infection and the upper genital tract pathology can be evaluated. Typical outcomes assessed include upper genital tract pathology by visual scoring for the presence of hydrosalpinx [12, 15], histological tissue evaluation [15], or maintenance of fertility following breeding [11, 13], all of which represent time consuming and/or invasive approaches for assessment of disease state. Imaging methods would allow for more rapid and noninvasive approaches for assessing disease pathology in mice and therefore may provide benefit for monitoring vaccine efficacy.

Magnetic Resonance Imaging (MRI) is an attractive method to investigate upper genital tract pathology or morphology caused by *Chlamydia* infection as it is completely noninvasive and provides excellent inherent soft tissue contrast, often without the use of additional agents. Further, the lack of ionizing radiation allows for repetitive longitudinal evaluation compared to other imaging techniques such as X-Ray, Computed Tomography (CT), and Positron Emission Tomography (PET). While MRI is considered a less rapid technique compared to Ultrasound (US), CT and Optical Imaging (OI), images of the entire abdomen or pelvis anatomy can still be obtained within minutes or even seconds.

In this study, we evaluated the potential use of MRI to assess the status of the genital tract in mice following infection with *C*. *muridarum*. MRI can be used to focus on detecting dilation/accumulation of fluid in the upper genital tract as a result of inflammation throughout the course of infection in mice. Further, we evaluated the potential impact of *Chlamydia* elementary body (EB) immunization on the status of upper genital tract pathology development as visualized by MRI. Together, these studies suggest that MRI is an attractive tool for assessing *Chlamydia* pathology and vaccine efficacy in animal models and could be beneficial for translating pre-clinical research into clinical evaluation of vaccine candidates.

## Methods

### Animal Husbandry

All animal experiments were approved by the Institutional Animal Care and Use Committee (IACUC), Merck Research Labs, West Point, PA. All procedures were performed in accordance with our institution’s IACUC guidelines in strict accordance with the recommendations in the Guide for Care and Use of Laboratory Animals of the National Institutes of Health.

Mice were housed in large mouse containers (n = 10 mice/box) with microisolator lids, and the rooms were maintained with controlled humidity and temperature, and 12 hour light-dark cycles. All containers contained nestlets and animals were provided standard chow (Purina 5001 rodent diet) and water *ad libitum*. The physical condition of the animals was monitored daily (7 days a week), and any health changes were noted.

At the end of the study, animals were euthanized using CO_2_ inhalation (10–30% CO_2_ in the air mixture inhaled per minute). For humane reasons, any animal identified as moribund and unable to move about or access food and water on the study were pre-defined to be sacrificed using the described CO_2_ inhalation. No animals were found to be moribund throughout the study.

### Generation of Infection

*Chlamydia* were propagated as described previously by Wooters et al [16]. Briefly, *C*. *muridarum* was obtained from Dr. Gerald Byrne (U. Tennessee, Memphis). HeLa 229 cells were used for propagation of *C*. *muridarum*. HeLa 229 cells were grown in Eagle's Minimal Essential Medium (EMEM; ATCC) supplemented with 10% heat-inactivated fetal bovine serum (FBS; Hyclone, Logan, UT), 50 μg/ml vancomycin (Sigma, St. Louis, MO), and 10 μg/ml gentamicin (Gibco; Carlsbad, CA). Host cells were seeded into tissue culture flasks at a cell density of 5 x 10^5^ cells/ml and incubated overnight at 37°C in 5% CO_2_ to achieve a confluent monolayer. Cell monolayers were infected with a chlamydial stock diluted in sucrose-phosphate-glutamate (SPG) buffer. The *C*. *muridarum* strain was cultured for 48 hours. *Chlamydia* were harvested from the infected cells by lysis and purified by centrifugation through 30% Renografin (Bracco Diagnostics, Princeton, NJ) and stored frozen at -80°C.

C57BL/6 mice were 6–8 weeks old at the start of the study (Charles River, Wilmington, MA). Thirty mice were randomly assigned into three groups. Groups of 10 mice each were either not challenged (control group), 10 mice received 3 prior immunizations with *Chlamydia* elementary bodies followed by a *C*. *muridarum* challenge (EB immunized-challenged group), and 10 naïve mice received a *Chlamydia* challenge only (naïve-challenged group). Progesterone [medroxyprogesterone acetate (Depo-Provera; Pfizer; New York, NY)] was administered subcutaneously (2.5 mg/dose) at 10 and 3 days prior to challenge or mock challenge (described below) for all three groups to synchronize the estrous cycle.

Each mouse in the EB immunized-challenged group was inoculated intraperitoneally (i.p.) with 1x10^5^ live *C*. *muridarum* inclusion-forming units (IFU) in sucrose-phosphate-glutamate buffer (SPG) at 60, 50, and 30 days prior to challenge. The challenge inoculum contained 30% Renografin purified *Chlamydiae* as described above. Mice were challenged intravaginally by direct instillation of 20 μl of SPG containing 1 x 10^4^ IFU of *C*. *muridarum* at day 0 (“*Chlamydia* challenge” for the EB immunized-challenged and naïve-challenged groups) or SPG buffer (“mock challenge” for the control group).

### Real-Time Quantitative Polymerase Chain Reaction

Real-time quantitative polymerase chain reaction (qPCR) was performed to confirm the presence of infection. Vaginal swab samples were collected at 7, 14, and 21 days post-infection and processed according to Wooters et al [16]. Briefly, DNA from genital swab samples (~100 μl) was extracted using the MagNA Pure 96 DNA and Viral NA small volume kit (Roche Diagnostics Corp., Indianapolis, IN) on a MagNA Pure machine according to the manufacturer's instructions.

The oligonucleotide primer set was designed for detection of all species of *Chlamydiae*. The sense primer, 16S DIR 5′-CGCCTGAGGAGTACACTCGC-3′, and anti-sense primer, 16S Rev 5′-CCAACACCTCACGGCACGAG-3′, were designed to amplify a 208-bp fragment of the *Chlamydial* 16S ribosomal subunit gene, conserved across *Chlamydia* strains and serovars. Primers were obtained from Sigma Genosys (The Woodlands, TX), and the probe, 16S Fam-5′-CACAAGCAGTGGAGCATGTGGTTTAA-3′ Tamra, was synthesized by Applied Biosystems, (Foster City, CA).

The 50-μL reaction mixtures consisted of 1× QuantiTect Multiplex PCR master mix without ROX (Qiagen USA, Valencia, CA), 100 nmol/L 16S probe, 200 nmol/L primer 16S DIR, 400 nmol/L primer 16S Rev, 30 nmol/L ROX reference dye, and 5 μL of sample DNA. Non-template controls consisting of the reaction master mix, primers, and probe, but no DNA, were included in each assay run. Reaction conditions were set as follows: 1 cycle at 95°C for 15 min, followed by 40 cycles at 94°C for 1 min and at 60°C for 1 min. Thermal cycling, fluorescent data collection, and data analysis were performed using the Stratagene Mx3005P system (Stratagene, La Jolla, CA) according to the manufacturer's instructions.

### MRI Acquisition

The ten mice from the three treatment groups (control, EB immunized-challenged, and naïve-challenged) were imaged prior to infection and at 7, 14, 21, 28, 40 and 63 days post-infection. MRI was performed on a 7T preclinical scanner (BioSpec 70/30 USR, Bruker, Billerica, MA) using a quadrature volume coil. Mice were anesthetized using 1.5–2.25% isoflurane by inhalation via tubing connected to the bite bar and were placed head first, supine into the magnet. Respiratory rate and body temperature were monitored during the course of the MRI exam using a small animal monitoring and gating system (SA Instruments, Model 1025, Stony Brook, NY); respiratory triggering was also utilized such that images were acquired at end expiration to minimize motion artifact. Mice were fasted overnight to minimize potential motion artifacts caused by peristalsis.

The MRI exam consisted of a series of localizers, followed by a 2D coronal T2-weighted fast spin echo acquisition (Rapid Acquisition with Refocused Echoes (RARE)). The field of view (FOV) of the coronal images covered the anatomy from the pelvic floor to the kidneys. In such T2-weighted (T2W) images, fluid appears hyperintense whereas tissue of more solid nature (e.g. muscle, liver) appears hypointense.

Imaging parameters for the T2W acquisition included the following: echo time (TE) = 20 ms, repetition time (TR) = 2500 ms, RARE partitions = 4, echo spacing = 10 ms, FOV = 4 cm x 3.2 cm, imaging matrix = 160 x 128 for an in-plane resolution of 0.25 x 0.25 mm/pixel, slice thickness = 0.50 mm with 0.55 mm gap between slices, 25–30 slices, flip angle = 90˚, 4 averages, and bandwidth = ± 25 kHz. Motion and fat suppression were also employed. The total scan time for each T2-weighted image was 5 minutes and 20 seconds prior to respiratory triggering delays, which was dependent on respiratory rate. Respiratory rate was typically between 30–70 breaths per minute.

### Visual Assessment of MRI Images and Ex Vivo Pathology

The coronal mouse pelvic and lower abdominal T2W images were visually assessed for structural or morphological changes in the mouse ovary/oviducts or uterine horn/uterus at baseline and each of the six timepoints post infection. Positive pathology was noted if the presence of excess fluid was seen in the ovary/oviducts or uterine horns/uterus; the presence of fluid was determined by comparison of MRI images of each animal at baseline and by comparison to the control, unchallenged group.

A scoring system for quantifying positive pathology from zero to four points was used, where one point each was used for the left ovary/oviduct, left uterine horn, right ovary/oviduct, and right uterine horn. One point was given for any positive pathology in any of the four regions of anatomy. For example, if both ovaries/oviducts contained hydrosalpinx, a score of two points would be assigned. If inflammation or fluid accumulation was found in all four regions, a score of four points would be assigned, and if no fluid accumulation was seen, a score of zero points would be assigned.

Animals were euthanized at Day 65 post infection using carbon dioxide asphyxiation. A gross pathological examination of the genital tract was performed at necropsy. Gross pathology waswere scored by visual evaluation for fluid accumulation and distended tissue compared with the unchallenged control group, and the same scoring system of 0–4 as described for the MRI images was used. Day 63 MRI findings were compared with *ex vivo* gross findings at necropsy, with the gross findings considered as the “gold standard.” MR images and gross pathology were separately and blindly scored by two independent scientists.

### Statistical Analysis

A linear mixed-effects model was used to characterize the mean profiles and variances of each of the three treatment groups [17]. For the MRI data, the changes in the scores from baseline were calculated for the naïve-challenged, control, and EB immunized-challenged groups. The differences from baseline within each treatment group, and the differences from baseline between each treatment group were compared. To compare the Day 65 gross necropsy scores with the Day 63 MR image scores, the differences between the two scores were calculated and statistically significant differences were determined using a linear mixed-effects model. A p-value less than 0.05 was considered statistically significant for all comparisons.

## Results

All animals survived to the end of the study, with no animals euthanized for humane reasons. No signs of illness were observedand all animals remained activewith no deaths prior to the endpoint of the study. At the end of the study, all animals were euthanized by CO_2_ asphyxiation.

### Generation of Infection

**Fig 1:**
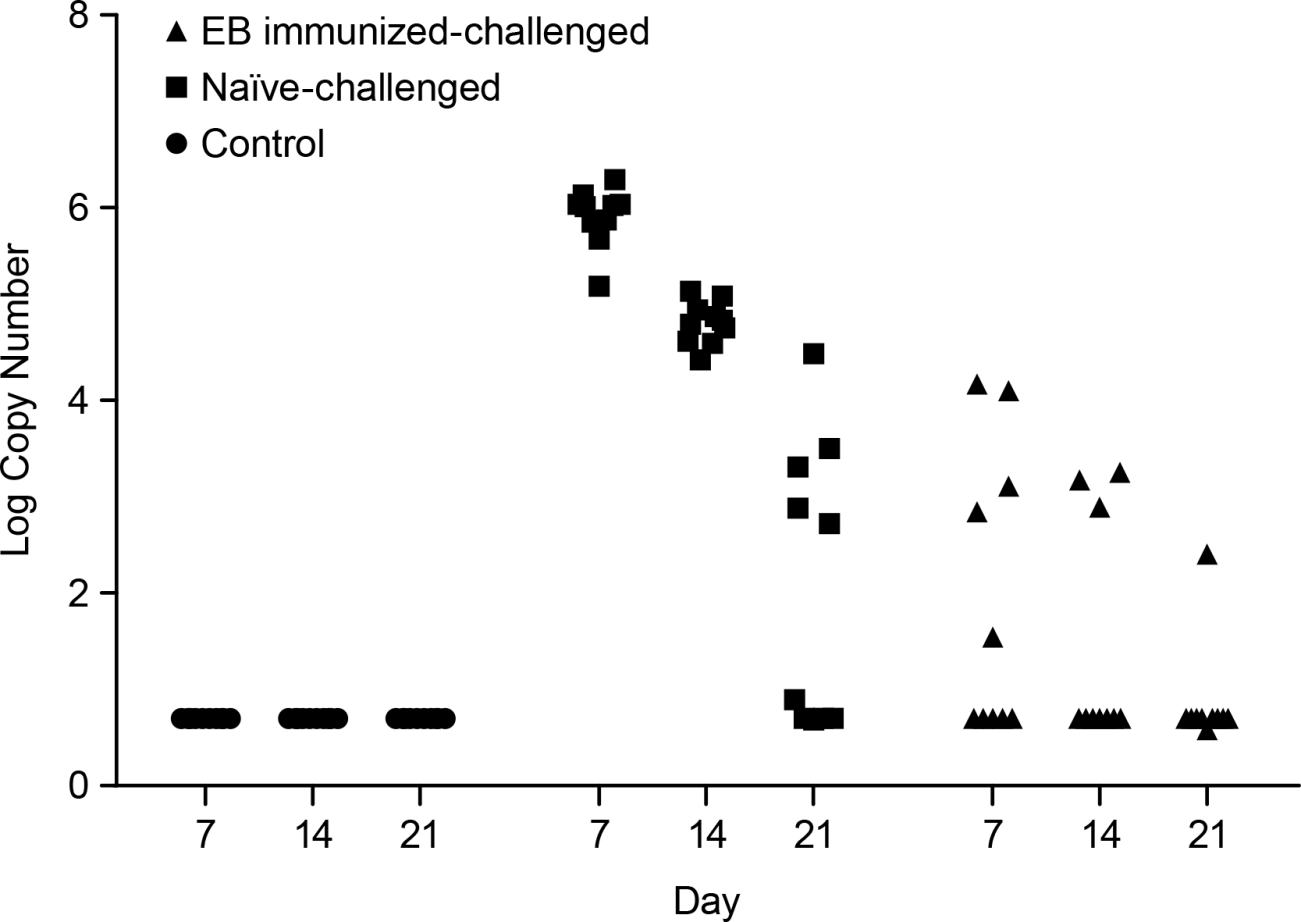
Bacterial counts recovered by vaginal swab. Summary of the course of infection over the first three weeks after challenge, as measured by qPCR.

The course of infection over the first three weeks after challenge is summarized in Fig 1. Upon qPCR evaluation of vaginal swab samples, 100% of the control mice were negative for *C*. *muridarum* infection at Days 7, 14, and 21. The EB immunized-challenged group had 50% of the mice infected at Day 7, 20% at Day 14, and 10% at Day 21. 100% of the naïve-challenged mice were infected at Days 7 and 14. At Day 21, 60% of the naïve-challenged mice were infected, consistent with the observation that lower genital tract *C*. *muridarum* infection has been shown to spontaneously resolve in mice over time [8, 9].

### MR Image and Gross Pathology Comparative Analysis

Table 1 summarizes the group mean and standard error of the mean (SEM) of the MR image scores for the three groups of mice during the course of the study.

**Table 1:** Mean and Standard Error of the Mean (SEM) of MRI Image Scores.

	Baseline	Day 7	Day 14	Day 21	Day 28	Day 40	Day 63
**Control**	0 ± 0	0 ± 0	0 ± 0	0.1 ± 0.1	0.2 ± 0.1	0.1 ± 0.1	0.3 ± 0.2
**EB immunized-challenged**	0.2 ± 0.1	0.6 ± 0.2	0.5 ± 0.2	0.7 ± 0.3	0.5 ± 0.2	0.5 ± 0.2	1.1 ± 0.2
**Naïve-challenged**	0 ± 0	0.4 ± 0.2	1.0 ± 0.2	1.1 ± 0.3	1.9 ± 0.3	2.7 ± 0.3	2.5 ± 0.3

**Fig 2:**
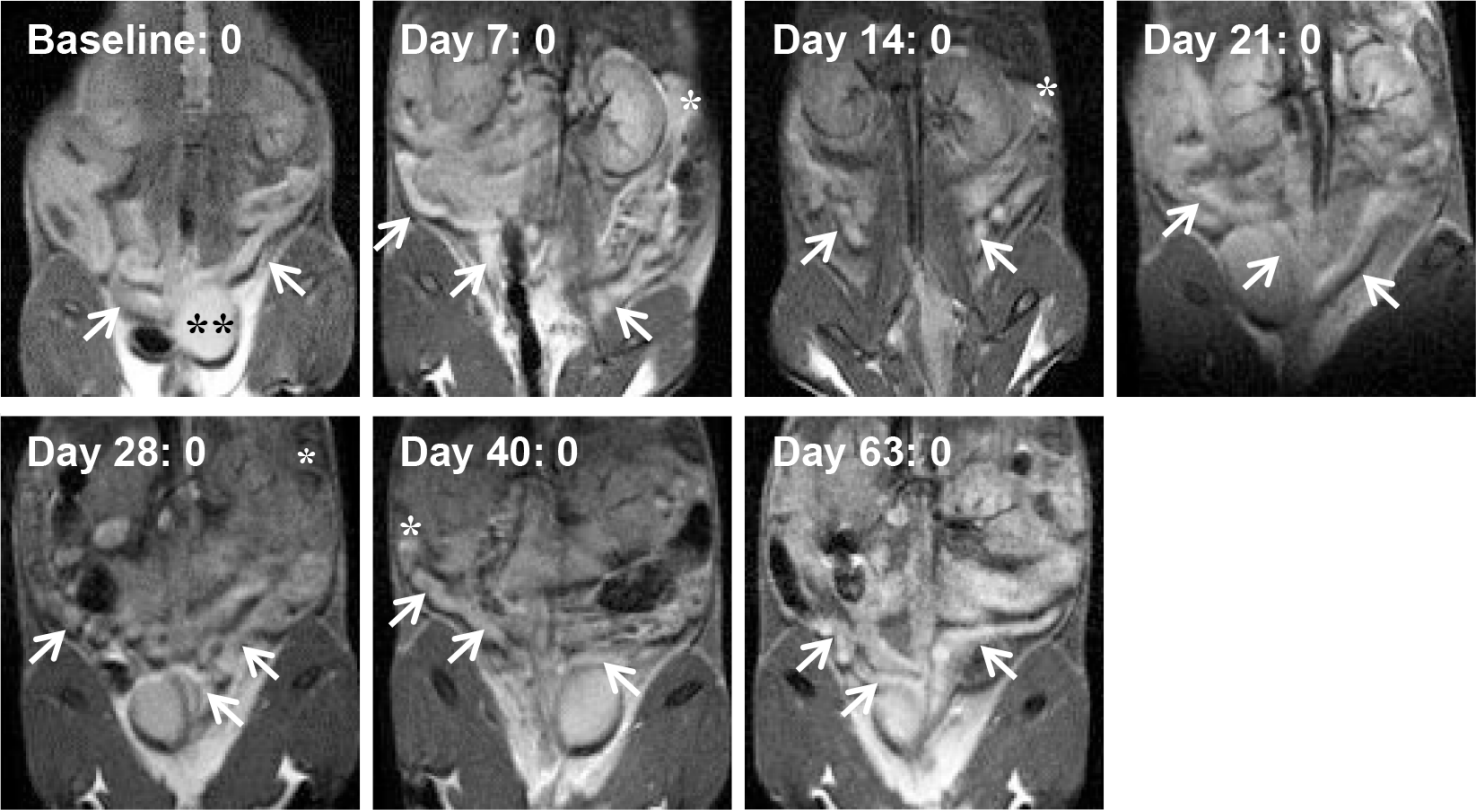
MRI images of an example control mouse at all time points during the study (n = 10 mice in control group). The coronal images are acquired such that the animal’s head is at the top of the figures, the tail at the bottom, and as if the animal is facing the viewer such that the animal’s left side is on the right side of the figures. Arrows point to uterine horns, asterisks denote an ovary/oviduct when present in the image plane, and the double asterisk identifies the bladder at baseline. Images are shown at the approximate plate of the uterine horn branching or to show both horns. The MRI image score is shown next to the time point, and the gross pathology score at Day 65 was 0 for this animal.

Fig 2 displays MRI images performed on one control mouse at each time point post-infection in the study to demonstrate an example of normal anatomy. The reproductive tract can be identified (arrows) and normal anatomy can be described. The fluid-filled hyperintense bladder can also be easily identified in this example (denoted with a double asterisk). As expected, the control group had consistently low scores throughout the study.

**Fig 3:**
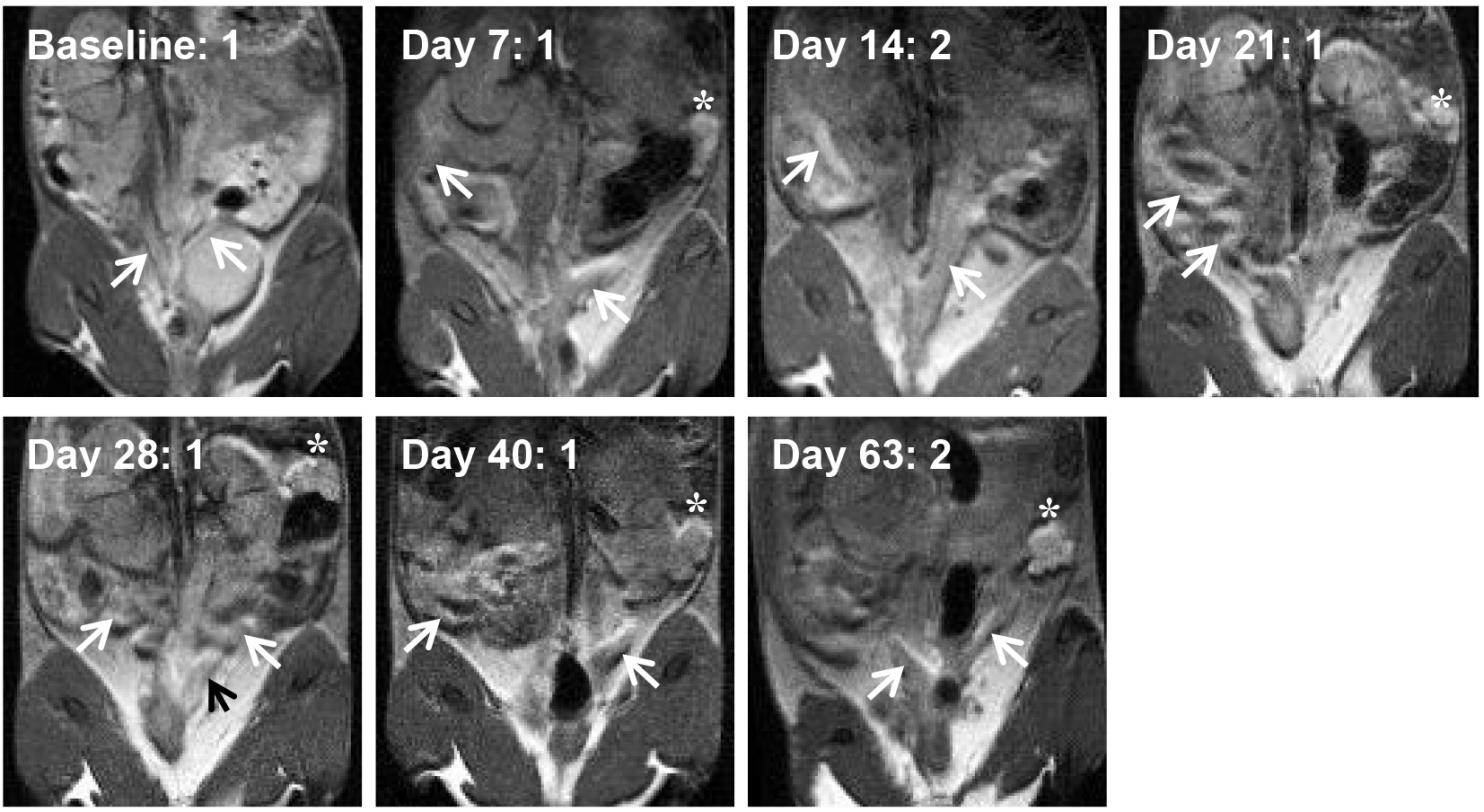
MRI images of an example EB immunized-challenged mouse at all time points during the study (n = 10 mice in EB immunized-challenged group). Arrows point to uterine horns, and asterisks denote an ovary/oviduct when present in the image. Black arrows are used only to provide contrast on hyperintense regions. Images are shown at the approximate plate of the uterine horn branching or to show horns. The presence of fluid in the animal’s left ovary/oviduct was seen from Day 21 through Day 63. The MRI image score is shown next to the time point, and the gross pathology score at Day 65 was 1 for this animal.

Fig 3 displays an example of one EB immunized-challenged mouse during the course of the study; slight pathology was seen throughout the study although overall scores were lower than the naïve-challenged group. Of note, the MRI image score for this animal was 2 at Day 14, dropped to 1 for Days 21–40, and then increased to 2 at Day 63; these scores were higher than average for the rest of the group, although the anatomy of the mouse can more easily be seen by readers. This variation in the scoring may be due to a possible clearing of the infection or an inflammatory response towards the beginning of the study and induced pathology at the end of the study, as mentioned in the Discussion.

The EB immunized-challenged group had slightly higher scores than both the control and naïve-challenged groups at baseline, which may be attributed to the earlier EB immunization. However, the EB immunized-challenged group scores remained low and relatively constant throughout the course of the study with scores similar to the control group. Comparison between MRI scores of the control group and the EB immunized-challenged group did not demonstrate statistically significant differences in any of the time points past baseline. At Days 7, 14, 21, 28, 40 and 63, the p-values were 0.29, 0.43, 0.29, 0.79, 0.59, and 0.11.

**Fig 4:**
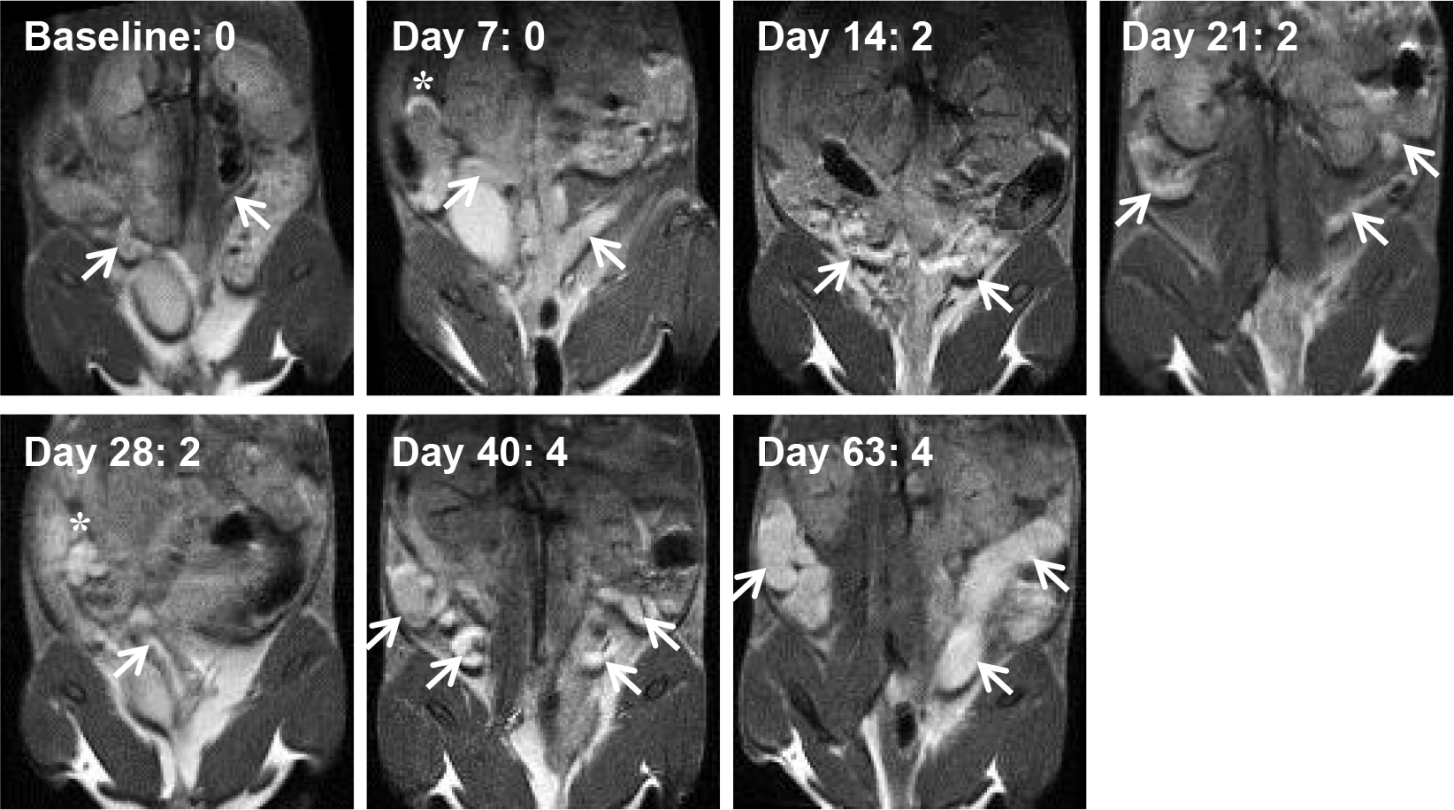
MRI images of an example naïve-challenged mouse at all time points of the study (n = 10 mice in naïve-challenged group). Arrows point to uterine horns, and asterisks denote an ovary/oviduct when present in the image. Images are shown at the approximate plate of the uterine horn branching or to show horns. Excessive fluid in the uterine horns is seen at Day 63. The MRI image score is shown next to the time point, and the gross pathology score at Day 65 was 3 for this animal.

The naïve-challenged group showed increasing scores from baseline, although by Day 28 the naïve-challenged group scores separated from the control and EB immunized-challenged groups and remained higher than the other two groups for the remainder of the study. To demonstrate the full time course and changes in pathological features of *Chlamydia* infection, Fig 4 displays one of the highest-scoring naïve-challenged mice from baseline to Day 63.

Comparison between the control group and the naïve-challenged group showed statistically significant increases in the naïve-challenged group beginning at Day 14 and continuing for the remainder of the study. No differences between the groups were seen at Day 7 (p = 0.29), although increases were seen at Day 14 (p = 0.02), Day 21 (p = 0.01), Day 28 (p < 0.001), Day 40 (p < 0.001), and Day 63 (p < 0.001). As such, the MRI scores could separate control and naïve-challenged mice by Day 14.

Comparison between the EB immunized-challenged group and naïve-challenged group showed no statistically significant differences at Day 7 (p = 0.99), Day 14 (p = 0.11), and Day 21 (p = 0.11). However, at Day 28, significant increases in the naïve-challenged group were seen (p < 0.001), and these increases were also observed at Day 40 (p < 0.001) and Day 63 (p < 0.001), indicating that the MRI image scores could separate EB immunized-challenged and naïve-challenged mice by Day 28.

Table 2 summarizes the incidence of pathology in the ovaries/oviducts detected by MRI images, and similarly, Table 3 summarizes the incidence of pathology in the uterine horns/uterus detected by MRI images. The incidence captured in Tables 2 and 3 counts the total number of pathologies per group of mice with a maximum score of 20 (10 mice/group and 2 potential areas (left and right) to contribute to possible pathology).

**Table 2:** Incidence of Positive Pathology in Ovaries/Oviducts Detected by MRI Images.

	Baseline	Day 7	Day 14	Day 21	Day 28	Day 40	Day 63
**Control**	0	0	0	1	1	0	3
**EB immunized-challenged**	1	3	4	4	4	3	8
**Naïve-challenged**	0	1	0	0	11	14	10

**Table 3:** Incidence of Positive Pathology in Uterine Horns/Uterus Detected by MRI Images.

	Baseline	Day 7	Day 14	Day 21	Day 28	Day 40	Day 63
**Control**	0	0	0	0	1	1	0
**EB immunized-challenged**	1	3	1	3	1	2	3
**Naïve-challenged**	0	3	10	11	8	13	15

### Ex Vivo Pathology

Table 4 summarizes the scores taken at the gross necropsy, as well as the incidence rate and location of positive pathologies. As expected, the control group scores remain the lowest of all the groups. No control animals showed any positive pathology. Slight increases were seen in the EB immunized-challenged group compared to the control group, although the naïve-challenged group had the highest scores at the end of the study.

**Table 4:** Mean and SEM of Gross Necropsy Scores by Group at Day 65.

	Day 65	Positive Pathology in Ovaries/Oviducts (Maximum of 20)	Positive Pathology in Uterine Horns/Uterus (Maximum of 20)
**Control**	0 ± 0	n = 0	n = 0
**EB immunized-challenged**	0.7 ± 0.3	n = 5	n = 2
**Naïve-challenged**	1.5 ± 1.3	n = 7	n = 8

**Fig 5:**
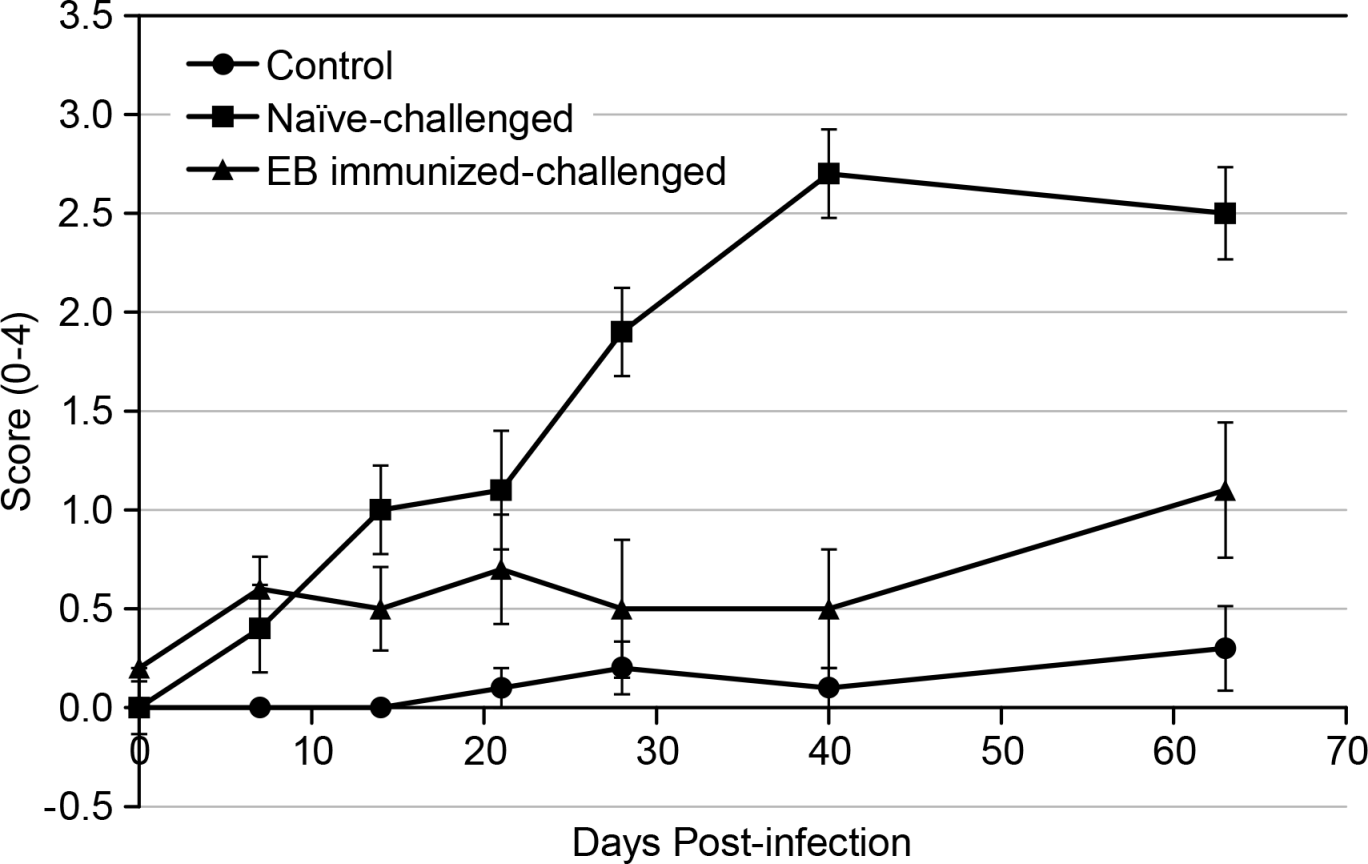
Group means of the MRI scores during the course of the study. Error bars represent the standard error of the mean (SEM).

**Fig 6:**
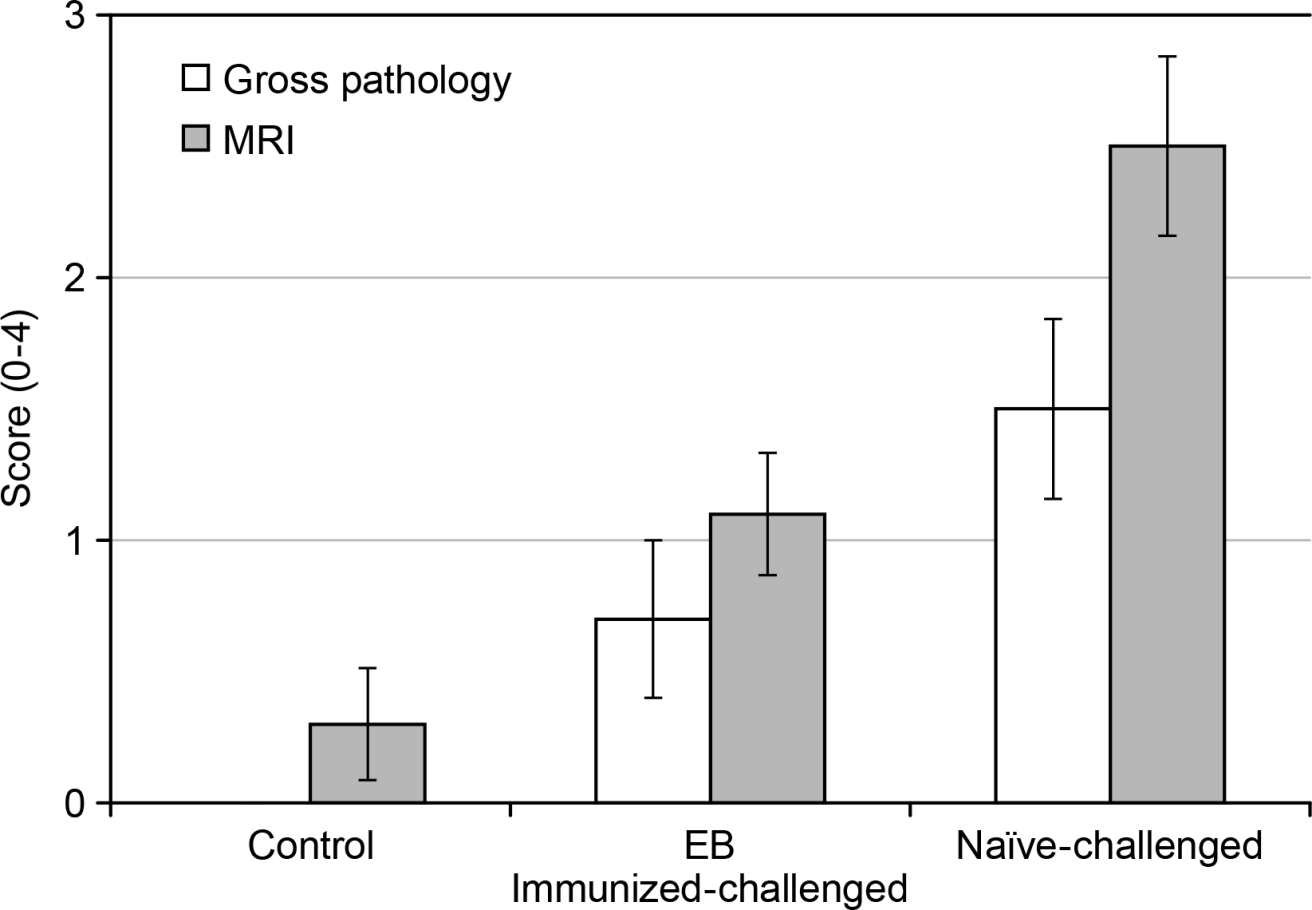
Comparison of the mean MRI image and gross pathology scores at the end of the study for the three groups of mice. MRI image scores at Day 63 were consistently higher than gross pathology scores at Day 65, although no significant differences were seen between the techniques for the control and EB immunized-challenged groups. Error bars represent SEM.

At the end of the study, the MRI image scores at Day 63 and the gross pathology scores were compared. Fig 5 plots the group mean and SEM of the scores as assessed by MRI images throughout the course of the study for all three groups of mice to demonstrate the time-course of infection and the MRI image scores. Similarly, Fig 6 summarizes the mean and SEM for each group as scored by gross pathology and MRI images to compare the different type of scores at the end of the study. Overall, the MRI image scores were greater than the gross pathology scores (p = 0.01), as seen, for example, in Figs 4 and 5. No significant differences were seen between gross pathology and MRI image scores for the control and EB immunized-challenged groups based on the fitted linear mixed effects model (p = 0.30 and 0.26, respectively).

## Discussion

In this work, we describe the application of MRI in mice for potential evaluation of disease pathology and morphology caused by *C*. *muridarum*. Using MRI, significant differences between control and naïve-challenged groups were identified at Day 14 and beyond, and between the EB immunized-challenged and naïve-challenged groups at Day 28 and beyond. No differences between the control and EB immunized-challenged groups were seen using MRI images throughout the course of the study. By demonstrating significant differences between the groups of mice, MRI demonstrated early specificity in identifying *Chlamydia* infection through the presence of fluid accumulation. MRI image evaluation also provided similar trends to gross necropsy evaluation at the end of the study. As such, MRI may be able to noninvasively assess the disease state caused by *C*. *muridarum* infection sooner than traditional assays, across the course of time following infection, and may be a potential future tool for evaluation of vaccines for *Chlamydia* in the mouse model.

To date, this work is the first known use of MRI assessing direct *C*. *muridarum* infection and EB immunized-challenged models in mice. Current evaluation of *Chlamydia* in mice requires time consuming and invasive methodology, which lack the ability for rapid screening and longitudinal evaluation. Noninvasive imaging techniques may improve this process.

Recently, Optical Imaging (OI) has been evaluated for invasive and noninvasive assessment *Chlamydia*-induced inflammation [18, 19]. OI is a molecular imaging technique used to detect disease processes in small animals, and near infra-red molecular imaging biomarkers have been used to detect inflammation in various animal models [20]. Using injected optical markers in conjunction with fluorescence molecular tomography and CT, in vivo measurements have quantified levels of inflammation in mouse models of atherosclerosis [21, 22]. However, OI is typically limited to early disease processes, such as acute inflammation, and does not provide anatomical information on late-stage processes such as pathology resulting from chronic inflammation.

The use of MRI in *Chlamydia* mouse models is also readily clinically translatable as 3D T2-weighted protocols are performed routinely on clinical pelvic MRI exams. Routine clinical female pelvic MRI is used to screen and evaluate benign pathologies of the uterus and fallopian tubes as well as evaluate and stage primary gynecological malignancies [23]. However, little research has been published using MRI to evaluate the pathology resulting from *Chlamydia* infection in the mouse or early Chlamydial infection pathology in patients apart from pelvic inflammatory disease [24, 25]. In a similar manner, these methods can also be adapted to evaluation in other large animal species that have been described for modeling of *Chlamydia* infection, such as non-human primates and pigs [9, 26–28]. Further, other preclinical disease states that result in fluid accumulation in the genital tract may benefit from using MRI, such as herpes simplex virus and gonorrhea, where fluid accumulation may be a potentially detectabable preclinical biomarker of inflammation.

The MRI image scores in the EB immunized-challenged group were greater than those observed in the control group at baseline and throughout the course of the study, although these differences were not statistically significant. We theorize that the increase in the MRI image scores for this group compared to the control group may be due to an inflammatory response caused by the EB challenge [29]. The inflammatory response may have resulted in the presence of additional fluid [30], which may have caused the MRI image scores to be higher as T2-weighted imaging is sensitive to the presence of fluid [31]. The MRI image scores for the EB immunized-challenged group also exhibited a higher level of variability across individual animals and time, as demonstrated by the mean MRI image score of 0.7 ± 0.3 at Day 21. This increase may reflect an active protective immune response with associated fluid increase during this time frame, which then resolved, resulting in significantly lower group MRI image score at the later time points compared with the naïve-challenged group (p<0.001 at Days 28, 40, and 63) [32].

Further optimization of the visualization of the anatomy can still be performed. Parts of the uterine horn can potentially be difficult to visualize as they appear strikingly similar to intestines, and visualization of the horns and ovaries/oviducts can be partially obscured by fecal pellets or shifted due to air pockets in the intestines. As such, regions of the genital tract may not be fully visualized and can easily be confused with other structures. This discrepancy between MRI and pathology can be potentially be improved by additional imaging of positive controls to better characterize common obstructions and observe the natural variability of features that can be seen in the mouse genital tract. Additionally, enhancements to the imaging paradigm can be performed to increase the resolution of the ovaries/oviducts, and better accommodate the respiratory and peristaltic motion artifacts and intestinal obstructions to more thoroughly visualize the tract and improve the accuracy of the MRI image scores. Future studies will need to further explore correlation of imaging data and gross pathology in the mouse model.

In conclusion, the utilization of in vivo MRI displays promise in assessment of urogenital morphological changes in response to *Chlamydia* infection as well as monitoring the efficacy of potential therapeutic treatments and vaccines. As mentioned earlier, the MRI evaluation showed similar trends to gross necropsy evaluation at the end of the study. As such, the MRI image scoring revealed better separation between the three groups of mice, which may be an advantage to traditional assays due to the ability to assess earlier time points and image the same mice longitudinally. These added benefits of MRI could allow for fewer numbers of animals to be used in studies, provide more humane non-invasive assessment, and enhance model refinement since the animals can be kept on study and be used to evaluate fertility.
